# A New Method of Dressing the Chest in Pneumonia, Pleurisy, Pleurodynia, Etc.

**Published:** 1891-12

**Authors:** 


### A NEW METHOD OF DRESSING THE CHEST IN PNEUMONIA, PLEURISY, PLEURODYNIA, ETC.

William Hunt (Annals of Gynecology and Poediatry, 1891,) advises the following method to dress the chest in a case of pneumonia, pleurisy, pleurodynia, etc.:

Do it on a large scale, in the same way that we now dress abrasions, bruises, etc.

If there is to be any cupping or other preliminary operation, have that attended to; then all the ingredients wanting are pure collodion and absorbent cotton, in smooth layers, and a good broad brush, like a mucilage brush.

Apply a very thin layer over the side affected, from spinal column to sternum, and secure it with collodion smeared thoroughly over it. Then go on with thicker layers, securing them with collodion until a good padding is obtained, paying particular attention to the edges. In double cases you can act accordingly. The advantages are:

1. The one dressing, if well applied, will last throughout the case ; thus

2. The fatigue and discomfort of frequent poulticing are avoided.

3. The side, in single cases, is held as in a splint, while the free side does the breathing. A firstclass non-conductor is covering the chest. It is possible that the contracting collodion may have some influence in controlling the blood supply.

4. There is no particular interference, in one who has a good ear, with physical examination. Maybe it would be a good thing if there was ; for having once made the diagnosis, what is the use of exhausting the patient every day by trying to find out whether one-eighth of an inch, more or less, is involved? The general symptoms will tell that.—*American Journal Med. Sciences.*

